# P10-04 Effects of a web-based versus a print-based physical activity intervention for community-dwelling older adults: Results of a randomized trial with a cross-over design

**DOI:** 10.1093/eurpub/ckac095.143

**Published:** 2022-08-29

**Authors:** Claudia Pischke, Claudia Voelcker-Rehage, Tiara Ratz, Manuela Peters, Christoph Buck, Jochen Meyer, Kai von Holdt, Sonia Lippke

**Affiliations:** 1 Centre for Health and Society, Medical Faculty, Heinrich Heine University Duesseldorf, Institute of Medical Sociology, Duesseldorf, Germany; 2 Department of Neuromotor Behavior and Exercise, Institute of Sport and Exercise Sciences, University of Muenster, Muenster, Germany; 3 Jacobs University, Bremen, Bremen, Germany; 4 Leibniz Institute for Prevention Research and Epidemiology, BIPS, Bremen, Germany; 5 Institute for Information Technology, OFFIS, Oldenburg, Germany

**Keywords:** Physical activity, older adults, eHealth, print-based intervention, web-based intervention, physical activity promotion, healthy ageing

## Abstract

**Background:**

Despite the crucial role of regular physical activity (PA) for preventing chronic non-communicable diseases, fewer than half of older adults in Germany engage in the recommended levels.

Objective: The aim of this study was to compare acceptance and effectiveness of two interventions for PA promotion among initially inactive community-dwelling adults aged 60 years and above in a nine-month randomized trial with a cross-over design.

**Methods:**

Participants were recruited offline and randomized to (a) a print-based intervention (PRINT n = 113) and (b) a web-based intervention (WEB, n = 129). Thirty percent (n = 38) of those in group (b) received a PA tracker in addition to WEB (WEB+, (c)). All intervention groups were offered ten weekly face-to-face group sessions led by trained student assistants. Afterwards, participants could choose to stay in their group or cross over to one of the other groups. Group sessions were continued monthly for another six months. Three-dimensional accelerometers to assess PA at baseline (T0), three-months (T1) and nine-months (T2) were employed. Intervention acceptance was assessed via self-administered paper-based questionnaires. Linear mixed models were used to calculate differences in moderate-to-vigorous PA (MVPA) between time points and intervention groups.

**Results:**

Of the initially recruited n = 242 participants, n = 91 (37.6%) were randomized to the WEB group, n = 38 (15.7%) to WEB+ and n = 113 (46.7%) to PRINT and n = 195 participants completed T1. Only n = 1 moved from WEB to PRINT and n = 15 from PRINT to WEB (WEB-WEB: n = 103, PRINT-PRINT: n = 76), when offered to cross over at T1. One-hundred and sixty participants completed T2. MVPA in min per day increased between baseline and T1, but these within-group changes in time disappeared after adjusting for covariates. MVPA decreased by 9 min/day between baseline and T2 (βtime = -9.37, 95% CI: [-18.58; -0.16]), regardless of intervention group (WEB vs. PRINT: βgroup*time = -3.76, 95% CI: [-13.33; 5.82], WEB+ vs. PRINT: βgroup*time = 1.40, 95% CI: [-11.04; 13.83]). Intervention acceptance was generally high.

**Conclusions:**

Despite high levels of acceptance of web- and print-based interventions for PA promotion and little movement between groups at T1, when given the choice, participation was not associated with increases in PA over time.

